# Targeted Therapy for Medullary Thyroid Cancer: A Review

**DOI:** 10.3389/fonc.2017.00238

**Published:** 2017-10-06

**Authors:** S. R. Priya, Chandra Shekhar Dravid, Raghunadharao Digumarti, Mitali Dandekar

**Affiliations:** ^1^Head Neck Surgery, Homi Bhabha Cancer Hospital and Research Centre, Visakhapatnam, India; ^2^Tata Memorial Centre, Mumbai, India; ^3^Medical Oncology, Homi Bhabha Cancer Hospital and Research Centre, Visakhapatnam, India; ^4^Head Neck Surgery, Paras Cancer Centre, Patna, India

**Keywords:** medullary thyroid cancer, targeted therapy thyroid cancer, vandetanib medullary thyroid, cabozantinib medullary thyroid, targeted radionuclide medullary thyroid, peptide receptor radionuclide therapy medullary thyroid, thyroid cancer recurrence, small molecule thyroid

## Abstract

Medullary thyroid cancers (MTCs) constitute between 2 and 5% of all thyroid cancers. The 10-year overall survival (OS) rate of patients with localized disease is around 95% while that of patients with regional stage disease is about 75%. Only 20% of patients with distant metastases at diagnosis survive 10 years which is significantly lower than for differentiated thyroid cancers. Cases with regional metastases at presentation have high recurrence rates. Adjuvant external radiation confers local control but not improved OS. The management of residual, recurrent, or metastatic disease till a few years ago was re-surgery with local measures such as radiation. Chemotherapy was used with marginal benefit. The development of targeted therapy has brought in a major advantage in management of such patients. Two drugs—vandetanib and cabozantinib—have been approved for use in progressive or metastatic MTC. In addition, several drugs acting on other steps of the molecular pathway are being investigated with promising results. Targeted radionuclide therapy also provides an effective treatment option with good quality of life. This review covers the rationale of targeted therapy for MTC, present treatment options, drugs and methods under investigation, as well as an outline of the adverse effects and their management.

## Background

Medullary thyroid cancer (MTC) which arises from parafollicular cells is less common than differentiated thyroid cancer (DTC) constituting between 2 (1) and 5% (2) of all thyroid malignancies. 13–15% of patients of MTC present with distant metastasis (DM) and have a 10-year survival of approximately 20% (3, 4). As with DTC, surgery is the treatment of choice (1). Unlike DTC, where the iodine avidity of follicular cells makes even metastatic DTC amenable to treatment, parafollicular cells do not concentrate iodine. Management options for residual, recurrent, or metastatic disease till a few years ago were re-surgery and/or radiotherapy (5, 6). Chemotherapy has been used with only marginal benefit (1). The quest to improve survival and quality of life (QOL) of patients with inoperable and/or metastatic MTC has resulted in significant research for other treatment modalities. The development of targeted therapy has brought a major advantage in the management of such patients. Two drugs, Vandetanib and Cabozantinib, have been approved for use in progressive or metastatic MTC (1). In addition, several drugs acting on other steps of the molecular pathway to MTC are being investigated with promising results. The application of targeted radionuclide therapy also provides an effective treatment modality with good QOL (1).

This review covers the rationale of targeted therapy for MTC, presents treatment options, drugs, and methods under investigation, and outlines the adverse effects and their management.

## Disease Outcomes

At presentation, 35–50% of patients with MTC have regional metastasis while 13–15% have DM mainly to the lung, bone, and liver (4, 7). An analysis of population-based (Surveillance, Epidemiology, and End Results or SEER) data revealed that among patients with tumors confined to the gland, the 10-year survival rate was 95.6%, whereas patients with regional stage disease had an overall survival (OS) rate of 75.5% (4). Patients presenting with DM at diagnosis had a 10-year survival of approximately 20% (4, 7). Machens et al. (8) corroborated calcitonin levels with prognosis and observed that calcitonin levels of over 150 pg/ml were associated with DM and extrathyroidal extension (in both familial and sporadic cases). Preoperative calcitonin levels of 500 pg/ml, nodal metastasis, and reoperative status best predicted failure to achieve biochemical cure (8). In high risk patients (residual disease, extrathyroidal extension, or nodal involvement), Brierley et al. (9) found that the locoregional recurrence free rate was 86% at 10 years with postoperative external beam radiation therapy (EBRT) as compared to 52% in those without adjuvant EBRT (*p* = 0.049). However, radiation may not confer survival advantage (6).

## Management of Recurrent or Metastatic Disease

### Locoregional Recurrence

Surgery remains the mainstay for operable locoregional recurrences (10, 11). At least a third of such patients achieve normalization of calcitonin levels (12) with long-term eradication of biochemical and radiological disease (13).

### Metastatic Disease

The liver is a major site for DM of MTC. Such patients are often symptomatic with pain and diarrhea. Surgical resection should be considered for amenable lesions to provide significant symptom-free survival (14, 15). For widespread unresectable lesions, radiofrequency ablation is an effective option with low morbidity (16). For diffuse lesions, transarterial chemoembolization of the liver achieves symptom control and partial response (PR) in a third of patients who have less than 30% of the organ involvement with intact liver and renal function (1, 17).

Lung metastases with associated mediastinal nodal disease should be evaluated for surgery if symptomatic and resectable (14). Alternatively, radiofrequency ablation maybe employed. Bone metastases usually occur in the vertebrae, pelvis, ribs, femur, skull, and humerus. If feasible, bony lesions should be resected for long-term symptomatic cure; postoperative radiation to the site of metastasis helps to ensure durable control (18). Radiofrequency ablation, vertebroplasty, or kyphoplasty along with judicious use of bisphosphonates are useful tools for palliation (19–21).

Single brain metastasis maybe resected if possible for palliation and to prolong survival (22). Multiple brain lesions not amenable to surgery must be considered for EBRT (1, 22).

Soft tissue metastases to sites, such as breast (23), testes (24), and skin (25), though usually amenable to excision or radiation, suggest disseminated disease and such patients usually succumb to disease in less than a year (1, 23–25).

In summary, small isolated metastases may be observed especially if the calcitonin has stabilized (26). For symptomatic progressive metastases not amenable local measures or where a single modality is insufficient for symptomatic relief, systemic therapy is preferred (11).

## Rationale for Targeted Therapy in MTC: Why and When

### Molecular Basis

Medullary thyroid cancer, whether inherited (MEN2 and FMTC) or sporadic, has a detectable association with mutations of the rearranged during transfection (RET) proto-oncogene which was described first in 1993 (27, 28). MTC is hereditary in 25% cases and occurs as a component heritable syndrome while it is sporadic in about 75% (29). RET is mutated in virtually all familial cases and in roughly 50% of sporadic cases (30, 31). Mutations usually occur in the cysteine-rich areas of exon 10 and 11 but may also occur in the tyrosine kinase domain (32).

Rearranged during transfection is classified as a proto-oncogene because a single activating RET mutation of one allele can lead to neoplastic transformation. Once activated, it transmits mitogenic and survival signals (33) *via* two major signaling cascades, namely RAS (34, 35) and the phosphoinositide 3-kinase (PI3K)/AKT/mTOR pathway (36). RAS and PI3K activate other signaling effectors proteins and secondary messengers influencing cell division, growth, and cell death (36), which in turn promote cell proliferation, invasion, and survival, eventually leading to MTC formation (36).

Rearranged during transfection encodes a plasma-membrane-bound receptor tyrosine kinase that is expressed in cells of neural crest and endodermal origin (37). Tyrosine kinases are enzymes that catalyze transfer of a phosphate residue from ATP to tyrosine residues in other proteins (substrates). Tyrosine kinases, which number about 90 in the human genome (38), play central roles in transducing extracellular signals (growth factors and cytokines) into activation of signaling pathways that regulate cell growth. Since a majority of oncoproteins are tyrosine kinases, the study of their inhibition is crucial (39, 40). Most inhibitors of tyrosine kinases receptors (TKRs) act at the intracellular ATP binding site and are low molecular weight inhibitors (in contrast to extracellular acting large molecular weight monoclonal antibodies), thus preventing phosphorylation and further action of the enzyme. Since TKRs are homologous, tyrosine kinase inhibitors (TKIs) inhibit several TKRs, and are, therefore, multikinase inhibitors, as opposed to certain monodrugs that focus on a single pathway such as mTOR/Akt, as described later.

Epidermal growth factor receptor (EGFR)-dependent RET activation: an interaction between EGFR and RET was recently described, with EGFR reported to be over-expressed in thyroid cancer cells (41). EGFR is a transmembrane TKR involved in the activation of MAPK and the PI3K/Akt pathway (42).

Recent lineage tracing (37) reveals that C-cell may have a shared origin from the primitive endoderm, a fact that may explain much of tumor behavior and secretory activity of MTCs. Consequently, expression of Fox-1 (forkhead box protein) is seen in invasive MTC as well as embryonal C-cells but not in differentiated normal C-cells or follicular cells. Thus, Fox-1 promoter can be a target for future research and targeted therapy (37).

Non-RET mutations: RAS family gene mutations (HRAS, KRAS, and NRAS) have been identified in 10–17% of cases of MTC and may be associated with a less aggressive behavior (43). RAS mutations may be exclusive of RET abnormalities (44). Genetic abnormalities found in other cancers, such as TP53, RB1, PIK3CA, and BRAF mutations, are rare in MTC (45).

Overexpression of vascular endothelial growth factor (VEGF)-2 receptors is described in MTC and is associated with increased metastasis (46, 47). Mutations in MET, a proto-oncogene encoding the receptor for hepatocyte growth factor have been reported in MTC (48). Another entity is the fibroblast growth factor receptor (FGFR), which is overexpressed in MTC; its inhibition is seen to decrease the proliferation of MTC cells (49).

All these pathways are potential targets for treatment of MTC. Drugs thus developed, focus on specific oncogenic proteins present in malignant but not normal cells, in contrast to chemotherapeutic agents (50). Further targeting of treatment as per the specific mutational profile may be possible, since there is mutational heterogeneity in MTC, not only between patients but also between tumor subpopulations as well as between primary and metastatic cells in a patient (51).

### Clinical Indications

Targeted therapy should be considered where other better tolerated and more accessible local treatments have been exhausted. Assessment of molecular markers calcitonin and carcinoembryonic antigen along with clinical and radiological evaluation helps to decide about when to consider use of these agents. In case of impending compression of specific sites such as the central nervous system or the airway, where a smaller disease volume may be critical, targeted therapy may be considered sooner (52). TKIs also help significantly in palliation of distressing secretory symptoms such as diarrhea and Cushing’s syndrome (53, 54) where they should be considered in adjunct to other palliative measures.

## TKIs for MTC

### “First Line” TKIs for MTC

Cabozantinib and Vandetinib are considered first line TKIs (Table 1). These drugs have been approved for use in MTC (11).

**Table 1:** Targeted drugs in use for medullary thyroid cancer (MTC).

S.No.	Name of drug	Pathway	Adverse effects	Partial response (PR)/progression-free survival (PFS)	Referenced trial year/phase/name
**Drugs approved for MTC**					
1	Vandetanib (64, 65)	RET VEGFR EGFR cKIT	Rash, nausea Hypertension Dry skin and mouth Headache Arrythmiarias Raised QTc interval Electrolyte disturbance	45%/30.5months	2011/III/ZETA TRIAL
2	Cabozantinib (57–61)	VEGF RET cKIT MET	Stomatitis hypertension Diarrhea Weight loss Hand and foot syndrome Thrombosis Osteonecrosis	28%/11.2 months	2012/III/EXAM TRIAL
3	Sorafenib (68–71)	RET VEGFR PDGFR FGFR cKIT	Hand–foot syndrome Diarrhea Weight loss Fatigue Skin rash Skin cancers	21%/18 months	2017/II
4	Sunitinib (72, 73)	RET VEGF PDGFR cKIT	Asthenia Mucosal and cutaneous toxicities Hand–foot syndrome Cardiac events	50%/16.5 months	2010/II
5	Pazopanib (74)	VEGFR FDGFR CKIT RET	Hypertension Diarrhea Fatigue LFT derangement	14.3%/9.4 months	2014/II
6	Lenvatinib (75)	VEGFR RET FGFR cKIT	Proteinuria Diarrhea Fatigue Hypertension Appetite weight loss Cough	49%/9 months	2010/II
7	Motesanib (52, 76, 77)	VEGFR PDGFR cKIT RET	Diarrhea Fatigue Hypertension Hypothyroidism Anorexia	0%/11 months Stable disease (SD) 81%	2009/II
8	Axitinib (78, 79)	VEGFR PDGFR cKIT	Fatigue Dyspnea Diarrhea Hypocalcemia Hypertension Hand–foot syndrome	0%/16.1 months Objective response rate (ORR) 35%	2008/II
9	Imatinib (80, 81)	RET cKIT PDGFR	Diarrhea Vomiting Abdominal pain Facial edema	0%/6 months	2007/II

#### Cabozantinib

Cabozantinib (XL 184) is an oral, small molecule pantyrosine kinase (RET, VEGFR, and cKIT) inhibitor (55). It was approved by the US Food and Drug Administration (USFDA) in November 2012 for metastatic MTC (56). In a randomized phase III trial (EXAM) (57, 58), 330 patients with progressive metastatic MTC were assigned to either cabozantinib (140 mg) or placebo once daily. A statistically significant prolongation in progression-free survival (PFS) was observed in the cabozantinib group. There was no difference in OS between the arms. The recommended dose is 140 mg daily (adjusted for tolerability). Adverse events (AEs) include stomatitis, hypertension and diarrhea, fatigue, weight loss, palmar-plantar erthyrodysesthesia syndrome (58, 59), uncommonly venous thrombosis and arterial thrombosis (59), rarely osteonecrosis (60), and fistula formation (61). Evaluation of a lower starting dose (60 mg/day) in a phase IV trial is ongoing (EXAMINER, NCT01896479) (62) along with trials for use in patients with renal (XL184-017) (63) and hepatic impairment (XL184-003) (63).

#### Vandetanib

This is a multikinase inhibitor, acting against RET, VEGFR, and EGFR. The U.S.FDA approved vandetanib for use in MTC in 2011, followed by approval in Europe in 2012. In a phase III (ZETA) trial (64), the efficacy and safety of vandetanib were compared with placebo in patients with locally advanced or metastatic MTC; 331 patients were randomized to receive vandetanib (300 mg/day oral) or placebo. The median PFS was 30.5 months in the vandetanib group vs 19.3 months in the placebo group (*P* < 0.001). No complete response (CR) was observed; however, PR was observed in 45% of the vandetanib group. Vandetanib was superior in all assessed subgroups (hereditary vs sporadic/unknown disease, prior systemic therapy vs no prior therapy). Frequent AEs were diarrhea, rash, nausea, hypertension, dry skin, dry mouth, and headache. QTc interval was prolonged in 15% subjects. Occasionally, cardiac arrhythmias, corneal opacities, hypocalcemia, hypokalemia, hypomagnesemia, and hypothyroidism were seen. Potentially treatment-related fatality occurred in five patients (2%) treated with vandetanib. An ongoing Phase IV trial is comparing 300 mg daily with 150 mg daily to understand the impact of dosing on response (NCT01496313) (65).

The decision between choice of drug, cabozantinib or vandetanib, must be made after considering the patient’s co-morbodities and the drug’s potential toxicities. A direct comparison between the two agents is not possible presently since the designs of the EXAM and the ZETA trials were different (66). It may be possible in the future to predict response to a TKI according to existing mutations to further individualize treatment (67). An outline of ongoing research on this aspect can be read later in this paper.

### “Second Line” TKIs for MTC

Other multikinase inhibitors active against MTC are described below (Table 1). These TKIs showed efficacy in Phase II trials and are considered as second line in patients where cabozantinib or vandetanib are not feasible (11).

#### Sorafenib

Sorafenib, a multitargeted kinase inhibitor targeting VEGFR, PDGFR, FGFR, c-KIT, BRAF, and RET, is approved for advanced radioiodine-refractory DTC in the USA and Europe. In a phase II trial (68), of 8 patients with MTC, an objective response rate (ORR) of 25% and disease control rate of 75% was observed with toxicities consistent with the known profile of the drug. A systematic review (69) examined the results of 8 phase II studies on the efficacy of sorafenib for all advanced thyroid cancers. The authors concluded that sorafenib at the starting dose of 400 mg twice daily led to PR in 22% of MTC patients which was more than that for differentiated and anaplastic cancers (21 and 6%). PD occurred in 6.5% of MTC patients (vs 21% of DTC). The commonest AEs seen with sorafenib are hand–foot syndrome, diarrhea, weight loss, and fatigue (69). An unusual adverse effect reported (though not in thyroid cancers) is sorafenib-induced skin cancers (70). A small study with DTCs by Chen et al. found that halving the dose of sorafenib reduces the rate of drug discontinuation due to AEs without affecting response (71).

#### Sunitinib

In a phase II study (72), 26 patients of advanced/metastatic MTC were administered 50 mg/day of sunitinib for 4–6 weeks. Median PFS and OS were 16.5 and 29.4 months. The AEs were asthenia, mucosal and cutaneous toxicities, hand–foot syndrome, and cardiac events (14%). A phase II trial (73) with a smaller dose (37.5 mg) resulted in 50% of patients showing PR but with significant AEs necessitating dose reduction in over half the subjects. Thus, existing data suggest that sunitinib has efficacy in MTC though not without adverse effects.

#### Pazopanib

Pazopanib, a multikinase inhibitor (potent action on VEGF 1–3, PGDF, FGFR, and milder action on RET) found to have activity against MTCs in preclinical studies, was studied in a multicentre international phase II trial in patients with advanced progressive MTC (74). Of 35 patients, PR was seen in 14.3% with a median PFS of 9.4 months and OS of 19.9 months. Nearly half the patients had received prior therapy with chemoradiation and or other TKIs (vandetanib, cabozantinib, and sunitinib). AEs were hypertension, fatigue, diarrhea, and deranged liver tests, necessitating discontinuation in three subjects. One therapy-related death was reported. Overall, Pazopanib may be considered in MTC patients refractory to other TKIs.

#### Lenvatinib

Lenvatinib is a multikinase inhibitor most active on FGFR, thus, targeting a pathway involved in resistance to VEGF inhibitors. In a phase II trial (75) enrolling 59 patients with advanced progressive MTC, an ORR of 36% was seen, both in patients with and without prior anti-VEGF therapy/conventional chemotherapy; the 6-month PFS was 67%. The commonest AEs were diarrhea, fatigue, hypertension, decreased appetite, weight loss, and cough; grade V toxicity occurred in 8.5% cases. AEs led to treatment withdrawal in a quarter of the patients. At present, its PFS and toxicity seems similar to the other agents described previously.

### Multikinase Inhibitors with Limited Efficacy

These are targeted agents that showed promise in preclinical or phase I or II trials but were not found efficacious enough to be used clinically for MTC.

#### Motesanib

Motesanib was the first TKI to be tested for MTCs alone. This is an oral small molecule TKI which is specific against VEGF 1, 2, and 3, besides wild-type RET and kit. The use of motesanib is based on the fact that VEGF is elevated upto 20 times in upto 75% of metastatic MTC (76). In a multicentre single arm phase II trial of 91 patients with advanced/metastatic MTC, the ORR was 2%, though 48% of the patients experienced prolonged stable disease (SD). The AEs though frequent were tolerable (52, 77).

#### Axitinib

Axitinib is a second generation VEGFR1–3 inhibitor and was evaluated in a phase II trial in 2008 (78), at a dose of 5 mg twice daily with 60 patients of advanced thyroid cancer including 11 MTC patients. PR was seen in two patients. On the other hand, a phase II trial (79) of patients with metastatic or unresectable TC, including six MTC patients recorded no PR to Axitinib, though SD was achieved in five cases for more than 4 months. The AEs of Locati et al. (79) Axitinib occur more often in the first year of treatment and rate of discontinuation due to AEs is comparable to that of other TKIs.

#### Imatinib

This prototype TKI was found to have limited inhibitory activity against RET; two trials included a total of 24 MTC patients who received imatinib. In these trials (80, 81), no objective responses were observed. Furthermore, treatment incurred significant toxicity.

#### Geftinib

Geftinib has not been found to be of appreciable efficacy for MTC. In a phase II trial for locally advanced or metastatic thyroid cancers, including MTC (82), there were no objective responses. There was tumor reduction in a third of the subjects though not enough to qualify as PR.

#### Ponatinib

Ponatinib is a newer multikinase inhibitor with exponentially higher activity against RET than other TKIs. Studies have proved its potential in MTC especially in cases where other treatment options had failed (83). However, observations in patients of chronic myeloid leukemia where it is used with good effect, showed a high risk of cardiovascular and peripheral vascular thrombosis (84, 85); this has prevented further development of this drug for MTC (86).

### Targeted Drugs in Development for MTC

#### Other Multikinase Inhibitors

Nintedanib (87), anlotinib (88), regorafenib (89), and sulfatinib (90) all multikinase inhibitors are being investigated in several phase II studies for use in MTC. Dinaciclib (91), a cyclin-dependent kinase inhibitor, has been seen to decrease thyroid cancer proliferation both *in vitro* and *in vivo*. It has not yet been investigated for MTC.

#### Farnesyl Transferase Inhibitors or FTIs (Tipifarnib)

A subset of MTC shows Ras mutations (43, 44). Inhibitors of farnesylation of Ras proteins lead to inhibition of coupling of these proteins with an isoprenyl group, blocking signal transduction and, thus, to cessation of growth (92). Of the three functional Ras genes, H-Ras, N-Ras, and K-Ras, the latter two can alternatively be geranylgeranylated, thereby escaping the inhibition of Ras activation (92). FTIs also exert their effect by inhibiting RET *via* the downstream MAPK pathway (92). The premise of their efficacy has been upheld in preclinical studies but has not been borne out in phase I, II, and III studies. In two Phase I studies (93, 94), tipifarnib (the most studied FTI) showed synergism when combined with sorafenib and showed sustained PR for patients of thyroid cancers including MTC. Tipifarnib as single agent is currently being investigated in a phase 2 trial in HRAS-mutated tumors, including MTC (NCT02383927) (95). Phase I studies showed myelotoxicity and neurotoxicity as rate limiting effects with other effects being rash, hand/foot skin reaction, fatigue, hypertension, anorexia, and diarrhea (96).

#### Repositioned Drugs

Cholesterol lowering agents (statins) such as lovastatin have anti-tumor efficacy in K-ras dependent thyroid tumors (97). This “repositioned” drug may be one more therapeutic option for MTC.

Histone deacetylase inhibitors (HDACIs) such as valproic acid (VA): histone proteins surround the DNA strands. Post-translational modifications occurring on the amino acid residues of these proteins modify chromatin structure and, thus, alter gene expression. Histone acetylation and deacetylation are the most well understood modifications. Histone deacetylases (HDACs) exert a pro-oncogenic effect by keeping genes involved in apoptosis in a transcriptionally quiescent state (98). HDAC inhibitors also induce G2/M arrest in neoplastic cells that do not have a functional G2 checkpoint and these cells, thus, undergo apoptosis. Since this effect is not seen in normal cells, HDACIs cause selective cell death of tumor cells (99). Though several HDACIs have not yet shown clinical benefit, these small molecule inhibitors are good potential therapeutic agents and under trial for all advanced thyroid cancers (100, 101). VA inhibits HDAC *via* its effect on Notch-1 signalling (102). Notch is an EGFR-like transmembrane receptor and an important regulator of ASCL1 gene expression and activity which in turn is essential for the normal and malignant development of thyroid C-cells (103). VA has efficacy on MTC cell lines as it restores Notch function, thus impeding tumor growth (104). Despite the strong preclinical evidence, VA has not been found to have *in vivo* efficacy against MTC except in combination with other drugs, such as lithium chloride (105).

### Monotherapy Agents and Epigenetic Therapy

#### Nelfinavir (NFV)

The heat shock protein (HSP) 90 chaperone is required for folding and stability of RET mutants (106). HSP90 overexpression is found in a significant proportion of MTCs and may correlate with metastases and *RET* mutations (107). HSP90 is a molecular target for the HIV protease inhibitor NFV. It has been shown to have wide spectrum activity *in vivo* against MTC cells and may emerge as an important therapeutic option. The adverse effects of NFV are seen to be similar to those of PI3–AKt pathway inhibitors such as everolimus (108).

#### mTOR Inhibitors

Everolimus is an mTOR inhibitor presently approved for treatment of neuroendocrine tumors and renal cell carcinoma. Since RET and RAS utilize the mTOR pathway, it follows that it is a potential subject for investigation for MTC. Two phase II trials (109, 110) conducted in patients with progressive metastatic or inoperable thyroid cancer, including MTC showed SD without any objective responses. However, combination of everolimus with sorafenib has shown promising results in the form of PR in 40% of the patients studied (111).

### Targeting Other Pathways

MKT-077, a rhodacyanin dye, accumulates in the cellular mitochondria of MTC cells and downregulates RET by direct and indirect pathways making it a potential therapeutic agent (112). cAMP analogs (8-Cl-cAMP) suppress *in vitro* MTC cell proliferation and may be of clinical use in the treatment of advanced MTC (113).

#### Combined Targeted Therapy

Certain small molecule inhibitors possess efficacy against MTC *in vivo* in combination with drugs of other groups (111). Methylating agent temozolomide along with chemotherapeutic agent capecitabine have a beneficial effect on metastatic MTC (114). Sunitinib and cisplatin cooperatively interfere with the autophagic lysosomal pathway and this combination appears to be a promising treatment option for metastatic or progressive MTC (115).

Certain combinations may be more potent than the individual agents alone (101, 105). Acquired resistance to targeted therapy, one of the mechanisms of which is aberrant DNA methylation, can possibly be overcome by employing a combination of targets such as with Everolimus plus 5-aza-2′-deoxycytidine (AZA), proven to be a highly synergistic combination even in Everolimus-resistant cell lines (116).

In a recent meta-analysis (117), the combination of cytotoxic chemotherapy and TKIs (vandetanib, cabozantinib, sorafenib, and sunitinib) was shown to have higher improvement in PFS (but not in OS) than chemotherapy alone in a variety of malignancies. This benefit, however, has to be weighed against AEs associated with this treatment.

## Pre-Empting and Managing Adverse Effects and Drug Interactions

Most series have documented significant AEs with targeted therapy (59, 64, 70, 85, 118, 119). Dose-dependent hypertension is a common occurence (120, 121) and guidelines (122) have been developed for its management including pretreatment monitoring of blood pressure, treatment of hypertension, and, if required, dose reduction. Cardiac effects (123–125) include prolonged QT interval (vandetanib, pazopanib), left ventricular dysfunction (pazopanib, axitinib), and congestive heart failure (sunitinib, pazopanib). Cardiac toxicity is often not predicted in preclinical studies (126), possibly due to unintended secondary targets of TKIs, toxic drug metabolites, and drug accumulation in the heart. The reversibility of trastuzumab-induced cardiac toxicity and the possibility of re-administering it to the patient have been mentioned (127). Though the same reversibility has not been described for TKIs in use for MTC, dose reduction, avoiding cardiotoxic drugs and reviewing drug combinations is important (128). Arterial thromboembolic events, such as cerebral infarction, cerebral ischemia, cerebrovascular accidents, myocardial infarction, and myocardial ischemia, are seen especially with panotinib (85) and cabozantinib (59) (upto 2%). Revascularization interventions may be indicated in severe cases (129).

Skin care in the form of protective shoes and use of urea-based cream prevents hand and foot syndrome. Avoiding exposure to soaps and direct sunlight is recommended. Supportive therapy, such as multivitamins, anti-diarrhoeals, dietary advice, and steroids for anorexia, prevents further complications. In view of TKI-induced hepatic AEs (130), pre-treatment baseline liver functions are mandated (131). TKIs are contraindicated in patients with transaminases more than 2.5 times normal in absence of liver metastases and five times in the presence of liver metastases. Development of grade III or IV hepatic toxicity during targeted therapy indicates the need for dose reduction or therapy discontinuation (131).

Drug interactions between TKIs and cytochrome enzyme inducers, such as ketoconazole (132) (alterations in either drug’s levels) and paracetamol (133) (additive hepatotoxicity), have been noted and accordingly the interacting drugs should be stopped or changed.

In view of risk of gastro-intestinal bleed and fistulization especially with VEGF inhibitors, it is important to avoid, stop, or change the therapeutic agent in patients with gastrointestinal bleed, immediately after injury or surgery, after radiation, in cases of impending fistulization or esophageal or tracheal rupture (134, 135). It is also a prudent precaution to avoid TKI treatment in cases of impending retinal bleed (131).

Squamous cancers of the skin which occasionally develop after TKIs (70) may be treated with wide excision (136). As these are unlikely to recur or metastasize, treatment discontinuation is not recommended (137). Sunitinib and sorafenib can lead to hypothyroidism due to decreased gastrointestinal absorption or due to diarrhea causing increased clearance. Monitoring of thyroid levels is, thus, recommended (138). Electrolyte imbalances also should be managed pre-emptively (137). Most of the AEs seen with targeted agents are class effects, that is, due to effect on target tissues, and thus may be unavoidable. There may also be a direct correlation between efficacy and AEs (139). Grade I or II AEs (as per Common terminology criteria—CTCAE) which can be managed with best symptomatic care do not necessitate dose reduction or discontinuation. However, in case of recurrent grade I or II and in case of more severe AEs (grade III or more), discontinuation ought to be considered (131).

### Improving Drug Selectivity—Individualization of Targeted Therapy

Consistent efforts are ongoing to correlate mutational status to drug efficacy and treatment decisions. Though important conclusions have been arrived at by different workers, there is still some way to go before definite algorithms can be formulated. The ZETA trial for Vandetanib in MTC (64) showed that patients with sporadic MTCs harboring a somatic *M918T* mutation had a higher response rate to vandetanib (54.5%; 55 of 101) than those without a somatic *M918T* mutation (32%; 33 of 103). In another study (140), 5 of 10 vandetanib-treated MTC patients showed PR, all harboring the RET918 mutation; also, mRNA expression of VEGFR was significantly higher in therapy responders. In another study, Kurzrock et al. found that there was no strict correlation between the RET/MET mutational status and clinical response of MTC to Cabozantinib (55). In a phase II trial of Sunitinib in MTC (141), PR or SD greater than 24 weeks was observed in 7 of 9 MTC pts with a M918T RET mutation. Three cell lines (142), respectively, expressing a C634W RET mutation, a M918T RET mutation, and a RET/PTC-1 rearrangement (in a case of DTC) were studied for response to 4TKIs; Cabozantinib was found to be the most efficient inhibitor for MEN2A derived cell lines, whereas vandetanib proved to be the most potent inhibitor for MEN2B. Carlomagno et al. found that the presence of Valine 804 mutation of RET as a structural determinant mediating resistance (143) while sorafenib has been found to have activity *in vitro* against the same V804 mutant (144). *In vitro* studies using cell lines with acquired resistance to vandetanib have shown persistent activation of the Ras/Raf/MEK pathway, which can be partly abrogated by sorafenib (145). In patients with *RET* wild-type tumors, it remains to be seen whether Ras mutations, identified in 60–80% of RET-negative sporadic MTC (35) have an impact on disease progression and if the therapeutic agent used needs to be one targeting Ras as well. In a phase II trial of lenvantinib, there was no difference in treatment response according to *RET* mutation status; however, high baseline levels of VEGF correlated with greater tumor shrinkage, and low levels of ANG2, sTie-2, HGF, and interleukin (IL)-8 were associated with tumor shrinkage and prolonged PFS (75). This distinction is important as the main serious AEs associated with the current RET TKIs are related to anti-VEGFR or anti-EGFR effects.

Thus, several possibilities for patients with different genetic profiles are gradually being discovered; and various workers continue to study and correlate genetic mutation status so as to determine the therapeutic option best suited to the individual patient.

### Resistance

Resistance to targeted therapy is well known and is also called the Escape phenomenon. Resistance may be innate or intrinsic or primary i.e., already present before targeted treatment is begun. *In vitro* studies show that the *RET V804M* (valine substituted with methionine) and *V804L* (valine substituted with leucine) gatekeeper mutations confer resistance to vandetanib (143). Although rare, these could result clinically in primary resistance to vandetanib. In patients with Ras mutations, identified in 60–80% of RET-negative sporadic MTC, intrinsic clinical resistance to vandetanib may exist (35). Intrinsic resistance also includes the ability of the tumor to take alternate and downstream signal pathways, such as Ras/MEK/EGFR (146). Secondary resistance develops after a variable period of definite response to TKI and it may be an On target or an Off target resistance.

On-target resistance is usually secondary but may be an unrecognized rapid cause of primary resistance. There is an alteration in structure or in activity of the target protein that results in reduced drug efficacy. This may be achieved by steric conformational change blocking access to the drug (147); by alteration in the topography of target site (148); or by alteration in ATP affinity (149) and last but not least by gene amplification (150), by which the number of copies of the target gene is increased many times over to enable the malignant cell proliferation to continue despite maximal binding of drug to target. Off target drug resistance is seen when the target oncoprotein stays inhibited and the cancer develops alternative sources of growth. It is termed as evasive or adaptive resistance when alternate pathways further downstream are activated such as FGRF/IL-8 (151); or other pro-angiogenic signaling agents are upregulated such as PGF/GCSF (152) or *via* negative feedback loops and paradoxical activation (153). Evasive resistance also occurs when there is recruitment of pro-angiogenic cells from the bone marrow; and when the tumor develops the capability to invade in absence of angiogenesis (in case of VEGF TKIs) (154). Finally, tumor cell signaling networks alterations and tumor microenvironment change which occur over time, influence disease progression as well as a drug’s effectiveness, thus resulting in secondary drug resistance (155).

### Predicting or Detecting Resistance

Pre-treatment prediction of efficacy of and resistance to a particular TKI, though not yet in routine practice, has been shown to be possible and feasible using cell lines (156) and patient-derived models (157). However, there are several caveats to these procedures and cautious interpretations have to be made. Serum calcitonin may act as a surrogate indicator of the development of resistance to the TKI being used with a study (158) demonstrating that an increase in serum calcitonin levels to greater than 40% may be used reliably as an early indicator of tumor progression or drug resistance and as a guide to consider change of treatment.

### Overcoming Resistance

Using a TKI that acts *via* more than one pathway is one way of countering resistance. This can be seen with cabozantinib that inhibits MET and VEGFR2 and effectively blocks the development of MET-driven evasive resistance, as opposed to agents targeting the VEGF pathway alone. Thus cabozantinib may provide a more sustained therapeutic effect (116, 159). Addition of an additional synergistic agent is another way of evading resistance. For example, cabozantinib induced upregulation of downstream factors, such as VEGF and MET, can be neutralized by adding 2-ME2 (2-methoxyestradiol) to MTC cell lines. This agent downregulates HIF 1α (hypoxia-inducible factor) expression and, thus, inhibits the abovementioned pathways (160). Similarly, chloroquine potentiates action of sunitinib (161) in cell lines by interrupting sunitinib-induced autophagic flux, thus causing further apoptosis in cancer cells.

### Conventional Cytotoxic Chemotherapy for MTC

Results with chemotherapy in MTC have been reported as equivocal, whether done with a single agent (dacarbazine or doxorubicin) (162) or as combination (usually dacarbazine along with 5FU/vincristine/doxorubicin/cyclophosphamide) (163, 164). Capecitabine, an oral prodrug of 5-fluorouracil and a reasonably safe option for disease stabilization in MTC (165) has been applied with good benefit in combination with another oral chemotherapeutic agent temozolomide (114). Conventional chemotherapy (with dacarbazine and its combinations) is currently recommended only for cases not eligible for, or resistant to tyrosine kinases (vandetanib or cabozantinib) (11). Cytotoxic agents have been found to act synergistically with small molecule TKIs for metastatic MTCs (115) and their role may re-emerge if these findings are substantiated.

## Targeted Radionuclide Therapy or Peptide Receptor Radionuclide Therapy (PRRT)

The molecular rationale for use of PRRT in MTC (or in other well differentiated neuroendocrine tumors) is the over expression of somatostatin type 2 receptors (SSTR) (166). Radiolabeled somatostatin analogs are agents used in diagnosis as well as therapeutics. Such an agent consists of a peptide somatostatin analog with high affinity to SSTR2 receptors (*viz*. octreotide), a radioactive component [yttrium (Y) or lutetium (Lu)] and an intervening chelator such as DTPA (diethylenetriaminepentaacetic acid). Y and Lu have properties suited to ablation of such tumors and act by direct effect and by anti-angiogenic effect (167). In earlier pilot studies (168) and in more recent trials (169), PRRT was found to be a viable therapeutic option with a beneficial effect on QOL (169). A phase II trial evaluating systemic 90Y-DOTATOC (tetraazacyclododecane-tetraacetic acid Phe-Tyr octreotide) treatment in patients with advanced MTC showed response in 9 out of 31 (29%) patients; there was improved survival in responders to treatment as compared with non-responders (i.e., 74.5 vs 10.8 months); however, 12.9% of patients developed hematological toxicity and 22.6% developed nephrotoxicity (170). Salavati et al. (171) treated over a period of 8 years, a cohort of 28 patients with recurrent and metastatic MTC. All patients had undergone previous treatment in the form of surgery, radiotherapy, conventional chemotherapy, and local palliative measures. Following confirmation of somatostatin expression using 68Ga-DOTATOC PET/CT, patients were treated with PRRT with 90Y or 177Lu-DOTATATE (DOTA-Tyr-octreotate). 17 patients (60.7%) showed SD, 5 of 28 (17.7%) showed PR and 6 (21.4%) showed disease progression. The median survival of patients with SD was 36 months, that of patients with PR was 72 months, and the median survival for patients with PD was 24 months. In a series of 265 patients (172) who underwent LuDOTATATE therapy for neuroendocrine malignancies, significant improvement in QOL scores was seen even in patients who showed no significant disease resolution; there was no case where QOL deteriorated to pre-treatment levels. PRRT has also been used in other neuroendocrine malignancies in the form of an infusion to selectively ablate liver metastases (173). Combination of PRRT with conventional chemotherapy has been found to provide benefit in neuroendocrine tumors (174, 175). Thus, PRRT promises to provide disease outcome benefits with improved QOL scores though the toxicity benefit ratio remains to be quantified (176).

A novel gene therapy technique has shown that MTC cells can be induced to take up iodine through calcitonin promoter-directed human sodium iodide symporter (hNIS) expression and, thus, can be ablated with Radioiodine (177).

## Conclusion

Inoperable recurrent and metastatic MTC needs intervention when symptomatic. Solitary, symptomatic metastases are best treated with surgery or radiofrequency ablation for clinical and biochemical benefit. The development of targeted therapy is the logical step forward when the disease becomes unapproachable with conventional treatment modalities. Evolution of knowledge about other molecular pathways that may be targeted and efforts to counter resistance by combining agents acting on separate points on the pathway is the rational next step. Though “personalization” of treatment according to the specific mutation or absence of it is still in the realm of investigation, categorization of patients as per treatment history, disease location, rate of progression and performance status is feasible; the emergence of guidelines concerning several aspects of treatment is evidence of effort in this direction.

## Author Contributions

SP, CD, and MD—design, collection of data, manuscript, editing, approval of final version, and accountability. RD—expert comments and approval of final version.

## Conflict of Interest Statement

The authors declare that the research was conducted in the absence of any commercial or financial relationships that could be construed as a potential conflict of interest.

## Abbreviations

DM, distant metastasis; AE, adverse events; HDACI, histone deacetylase inhibitor; VA, valproic acid; QOL, quality of life.
